# ΔCt-informed, calibrated logistic regression accurately attributes *mecA* in *Staphylococcus aureus*-positive wound specimens

**DOI:** 10.1128/spectrum.03230-25

**Published:** 2026-03-31

**Authors:** Mehdi Dehghani, Hans Norouzi, Shabnam Dehghan, Keagan H. Lee, Howard L. Martin

**Affiliations:** 1 Sagis Diagnostics, Houston, Texas, USA; 2 BioExcel Diagnostics, Houston, Texas, USA; The University of Arizona, Tucson, Arizona, USA

**Keywords:** wound, PCR, culture, MRSA, methicillin

## Abstract

In wound specimens, co-detection of *mecA* and *Staphylococcus aureus* by PCR does not necessarily indicate methicillin-resistant *S. aureus* (MRSA) because coagulase-negative staphylococci (CoNS) frequently harbor *mecA*. We evaluated a ΔCt-informed, biologically gated, calibrated logistic regression model to attribute *mecA* to *S. aureus* versus CoNS. Using paired culture/antimicrobial susceptibility test and multi-target real-time PCR cycle-threshold values (internal *n* = 93; external *n* = 47), we trained fivefold cross-validated models in the culture-positive *S. aureus* subset (*n* = 36) and applied an *S. aureus* PCR gate (no attribution when *S. aureus* PCR is negative). The primary model achieved sensitivity 90.9% and specificity 92.0% for MRSA attribution with AUC 0.931 (out-of-fold). Decision curve analysis showed positive net benefit across clinically relevant thresholds; at the prespecified 50% cutoff, the model achieved a net benefit of 0.222 compared with negative benefit for a treat-all strategy. In an external cohort, *S. aureus* detection by PCR versus culture showed 92.3% sensitivity and 97.1% specificity; within *S. aureus* PCR-positives (*n* = 12), MRSA attribution reached 100% sensitivity and 87.5% specificity (accuracy = 91.7%). This framework improves *mecA* interpretability in polymicrobial specimens tested by panel-based molecular assays and can reduce unnecessary MRSA-directed antibiotics.

**IMPORTANCE:**

This work addresses a practical diagnostic gap in routine wound infection testing using panel-based PCR. In some CAP/CLIA laboratories using multiplex or panel-based PCR, detection of *Staphylococcus aureus* and *mecA* does not reliably resolve whether methicillin resistance originates from *S. aureus* or coagulase-negative staphylococci in polymicrobial specimens. Our approach is not a stand-alone methicillin-resistant *S. aureus* (MRSA) screen; it functions as a post-analytic attribution layer that probabilistically disambiguates the source of *mecA* within existing PCR workflows. By leveraging routinely available cycle-threshold data within a biologically gated logistic framework, this method provides transparent and auditable MRSA/methicillin-susceptible *S. aureus* attribution without additional instrumentation or workflow changes. Accurate attribution reduces biologically implausible MRSA calls while preserving detection of probable MRSA, supporting more rational antimicrobial stewardship in polymicrobial wound infections.

## INTRODUCTION

Wound infections are often polymicrobial, commonly involving pathogenic *Staphylococcus aureus* and commensal coagulase-negative staphylococci (CoNS) (1, 2). *S. aureus* is a major cause of invasive infections, whereas CoNS, particularly *Staphylococcus epidermidis*, frequently colonize chronic wounds and indwelling devices. In these contexts, CoNS contribute to biofilm formation and act as reservoirs of antibiotic resistance determinants, such as *the mecA* gene (3, 4). The presence of the *mecA* resistance gene complicates diagnostic interpretation in such polymicrobial settings. Because *mecA* encodes the penicillin-binding protein 2a (PBP2a), which confers methicillin resistance, its detection in an *S. aureus*-positive specimen may indicate methicillin-resistant *S. aureus* (MRSA). However, CoNS commonly harbor *mecA* on *staphylococcal cassette chromosome mec* elements, so assuming that *mecA* must originate from *S. aureus* can be misleading and can prompt unnecessary use of antibiotics such as vancomycin or linezolid, instead of β-lactams (e.g., amoxicillin–clavulanate) when the infection is methicillin-susceptible. Such unwarranted treatment escalations increase drug toxicity, cost, and sometimes complicate treatment strategy (1, 3, 5). These insights underscore the need for diagnostic strategies suited for polymicrobial samples when molecular assays cannot link *mecA* to CoNS or *S. aureus*. To address this issue, previous studies used ΔCt (*S. aureus* cycle threshold [Ct] – *mecA* Ct) thresholds to determine the connection between these two targets. For instance, the MRSA/SA ELITe MGB kit considers a small difference (ΔCt < 2 cycles) between *mecA* and *S. aureus* signals as indicative of MRSA. In contrast, larger differences imply methicillin-susceptible *S. aureus* (MSSA) or mixed populations (6). Another approach, combining selective broth enrichment and real-time nuc–*mecA* duplex PCR, achieved a sensitivity of 93.5% and specificity of 88.6% for MRSA detection (7).

Although these stand-alone MRSA assays exist and have utility in nasal screening or targeted surveillance, they are not optimized for polymicrobial wound specimen testing. In contrast, multiplex or panel-based molecular assays, including laboratory-developed tests, are increasingly used in high-complexity clinical laboratories to report multiple pathogens and antimicrobial resistance genes from a single specimen, and similar approaches have been applied to wound and soft-tissue infections. Our approach was designed to enhance these multiplex workflows by refining the attribution of *mecA* signals within the panel, rather than relying on a separate single-target assay. By embedding ΔCt-derived features and biologic gating into the panel-based molecular testing workflow, we improve the interpretability of resistance gene results without altering the established analytical workflows, enabling attribution of *mecA* to *S. aureus* versus CoNS in wound infection specimens.

## MATERIALS AND METHODS

### Study design and ethics

This diagnostic accuracy study (Fig. S1) followed the Standards for Reporting of Diagnostic Accuracy Studies guidelines (8). We analyzed an anonymized data set that was previously utilized in the study titled “Comparative Diagnostic Evaluation of Real-Time PCR and Culture for Detecting Pathogens in Podiatric Wound Infections” (9). The study involved paired aerobic culture and antimicrobial susceptibility testing (AST), conducted by a reputable commercial reference laboratory, along with a probe-based Wound PCR panel (BioExcel Diagnostics). Since the data set was fully de-identified, the analysis was exempt from human-subjects research regulations under 45 CFR 46.104. The internal cohort (*n* = 93) consisted of real-world, provider-ordered wound specimens, predominantly derived from podiatric and soft-tissue infections, including diabetic foot ulcers, chronic wounds, and other clinically infected wound sites, as previously described (9).

For all 140 wound specimens included in this analysis (93 internal, 47 external), paired swabs were collected from the same wound site during the same clinical encounter: one specimen was submitted for routine aerobic culture with AST, and the other was submitted to the BioExcel Diagnostics laboratory for multiplex PCR testing. Thus, timing, collection site, and specimen handling were equivalent across both index and reference tests, and any differences in turnaround time reflected laboratory workflow rather than differences in specimen acquisition.

The external cohort (*n* = 47) also represented an independent set of provider-ordered clinical wound specimens collected using the same paired-swab workflow and submitted concurrently for routine culture/AST and PCR testing. As in our prior wound infection study, both PCR and culture were requested simultaneously by the treating provider as part of routine diagnostic practice. These specimens were not used for model training or cross-validation (CV) and were included to assess the generalizability of the ΔCt-informed attribution framework in a distinct real-world sample set. Because specimens were collected from clinically infected wound surfaces using standard swab-based sampling, polymicrobial mixtures and background skin flora, including coagulase-negative staphylococci, were expected and reflect routine clinical practice rather than curated research sampling.

### PCR assays and feature derivation

To ensure standardized input for downstream logistic regression modeling, we utilized real-time PCR data previously generated in our comparative evaluation of PCR and culture for wound infections (9). In that study, bead-based mechanical lysis was used to disrupt both gram-positive and gram-negative organisms, followed by extraction with the MagMAX Microbiome Ultra Nucleic Acid Isolation Kit (Thermo Fisher Scientific). Amplification was performed on the SmartChip Real-Time PCR System (Takara Bio) using TaqMan probe-based assays (Thermo Fisher Scientific) targeting a broad panel of clinically relevant pathogens and antibiotic resistance genes. Each run incorporated internal controls for extraction efficiency, amplification performance, and contamination, along with positive and negative assay controls.

From this data set, real-time PCR assays provided Ct values for *S. aureus*, *Staphylococcus lugdunensis*, CoNS, *Staphylococcus saprophyticus*, and *mecA*. Targets were considered positive if Ct ≤ 34. Raw Ct values for the *mecA* assay, *S. aureus*, and CoNS were recorded. Detection of CoNS was performed using a pan-CoNS real-time PCR assay (Ba07921963_s1; TaqMan probe-based assay, Thermo Fisher Scientific), designed to detect multiple CoNS species, including *Staphylococcus epidermidis*, *Staphylococcus haemolyticus*, *Staphylococcus warneri*, *Staphylococcus lugdunensis*, and *Staphylococcus saprophyticus*.

For each specimen, we calculated the gap between *mecA* and each organism as *dCt_S.aureus_ = (Ct_mecA_* − *Ct_S.aureus_*) and *dCt_CoNS_ = (Ct_mecA_* − *Ct_CoNS_*). When direction was not needed, the absolute output values for these equations (*adCt*_*S.aureus*_ or *adCt*_*CoNS*_) were used. To quantify the closeness of *mecA* to each organism’s Ct value, we applied a logistic transform to the calculated absolute gaps using the equations below:


$$P_{S.aureus}=\frac{1}{\left(1+\exp\{2adCt_{S.aureus}-2\right\})}\;and\;P_{CoNS}=\frac{1}{\left(1+\exp\{2adCt_{CoNS}-2\right\})}$$

These *P*-values were only defined when the corresponding Ct pair existed; otherwise, they were set to 0. ΔCt features were omitted if a component’s Ct was unavailable; sentinel Ct values (e.g., 40) were not employed for non-detects.

### Culture and AST data

Review of available microbiology reports showed that methicillin resistance status for *Staphylococcus aureus* was determined using standard-of-care phenotypic culture and AST performed by a reputable commercial clinical microbiology reference laboratory, as described in “Study design and ethics,” above. The reports explicitly documented oxacillin susceptibility testing, including MIC determination and categorical interpretation. Because AST was performed as part of routine reference laboratory workflows and this analysis was conducted retrospectively, platform-specific AST metadata (e.g., automated broth microdilution systems versus disk diffusion methodologies) were not consistently available in the accessible reports and were therefore not specified.

### Machine-learning model and calibration

The primary classifier was logistic regression with class weighting (class_weight = “balanced”). For linear models, we applied median imputation, followed by standardization and classification using scikit-learn pipelines. Median imputation was performed only on the training data. The pipeline included median imputation for missing values, feature standardization, and Platt probability calibration via “CalibratedClassifierCV (method = “sigmoid,” cv = 3)” inside each training split; the calibrated model then generated predictions for the held-out fold. Unless otherwise stated, decisions used a fixed threshold of 0.50 on calibrated probabilities. Fivefold stratified cross-validation generated out-of-fold (OOF) probability predictions (10–12). Model training and cross-validated evaluation were conducted in the 36 culture-confirmed *S. aureus*-positive cases (reference cohort). After CV, we fit the final calibrated model on all 36 specimens and generated predictions for the full 93-specimen data set and eventually the external validation cohort. A biologic plausibility gate was applied post-prediction (never during CV or training). If PCR was negative for *S. aureus*, the MRSA probability was set to 0, and the qualitative output reported “No MSSA/MRSA.” When PCR was positive for *S. aureus*, we applied the 0.50 threshold to classify MRSA vs MSSA. External-cohort MRSA/MSSA attribution followed the same rule (classify only when PCR was positive for *S. aureus*).

Comparator models, linear SVM, random forest, and HistGradientBoosting, were trained on the same feature set with similar imputation methods; linear SVM used scaling and class weights, random forest used class_weight = “balanced_subsample,” and HistGradientBoosting used only imputation. Class imbalance (25 MSSA versus 11 MRSA in the reference cohort) was addressed through stratified data splits and class weighting.

### Decision curve analysis

Decision curve analysis was performed to evaluate the clinical utility of the calibrated logistic regression model across a range of probability thresholds. Net benefit was calculated as Net benefit = (*TP* / *N*) − (*FP* / *N*) × (*p_t_* / (1 − *p*_*t*_)), where *p_t_* represents the threshold probability at which a clinician would act as if MRSA were present. A threshold of *p_t_* = 0.50 was prespecified as a neutral and interpretable reference point for performance reporting and confusion-matrix–based metrics. This threshold does not represent an optimized or universal clinical decision boundary; it was used to provide consistent estimates of sensitivity, specificity, and accuracy. In decision curve analysis, relative harm weighting between false MRSA and false MSSA outcomes is implicitly encoded by the threshold probability. At *p_t_* = 0.50, false-positive and false-negative outcomes are weighted symmetrically, whereas lower or higher thresholds reflect greater relative concern for missed MRSA or unnecessary MRSA-directed therapy, respectively. At *p_t_* = 0.50, the implied harm ratio *FP*:*FN* equals *p_t_* /(1 − *p_t_*) = 1, so *FP* and *FN* are weighted equally.

### Performance metrics

Primary metrics that were evaluated are sensitivity, specificity, accuracy, ROC AUC, and Brier score, computed from OOF predictions. Calibration was assessed with reliability curves and expected calibration error. Uncertainty for performance estimates was summarized with non-parametric bootstrap resampling; additional robustness checks included per-fold summaries, 100 × 5 repeated cross-validation, and delete-1 jackknife influence (see Results). The Python code employed for the analysis is provided in the supplemental material.

### Index test and blinding

All data outputs presented in this study were generated using a Python analysis pipeline (mrsa_audit_pipeline.py) without any manual adjustment, subjective interpretation, or retrospective reclassification of calls. For the internal cohort, although this was a retrospective data set, performance metrics were calculated from out-of-fold predictions in cross-validation; thus, each case’s prediction was generated by a model that had no access to its own reference standard. For the external cohort, the trained model produced predictions without access to culture or AST results, which were only used for performance comparison.

## RESULTS

### Data set composition

This study analyzed 93 wound and soft tissue infection specimens that were previously included in our comparative diagnostic evaluation of real-time PCR and culture for podiatric wound infections (9). In that study, PCR demonstrated 97.2% sensitivity and 94.7% specificity for detecting *S. aureus*, underscoring the robustness of the PCR workflow for detecting *S. aureus* in podiatric wound specimens (9). Within the present data set (Table 1 and Tables S1 and S3), 38 of 93 specimens (40.9%) were PCR-positive for *S. aureus*. Among these, 22 also demonstrated concurrent CoNS detection using a Ct ≤ 34 threshold, underscoring the challenge of attributing *mecA* to *S. aureus* versus CoNS in polymicrobial wound specimens. *mecA* was detected in 28 of the 38 *S. aureus* PCR-positive specimens, and 19 specimens were simultaneously positive for *S. aureus*, *mecA*, and CoNS. Within this overlapping subset, 8 of 19 specimens were classified as MRSA by the model, with discordant MRSA attribution limited to 2 of 19 specimens in the internal cohort. This overlap provided the rationale for deriving ΔCt-based features and developing a probabilistic logistic regression model to assign *mecA* to its most likely staphylococcal source. Culture identified *S. aureus* in 36 of 93 specimens, including 25 MSSA and 11 MRSA. Among the 11 culture-confirmed MRSA specimens, 10 were PCR-positive for *S. aureus*, while one MRSA specimen was PCR-negative. These 36 culture-confirmed cases comprised the reference cohort used for model training and evaluation.

**TABLE 1:** Target-level prevalence in 93 wound specimens^*a*^

Target	Count	Percent
CoNS	61	65.6%
*S. aureus*	38	40.9%
*mecA*	62	66.7%
*S. lugdunensis*	11	11.8%

^*a*^ Prevalence of staphylococcal targets by probe-based real-time PCR in wound specimens. Percentages are expressed relative to 93 total specimens.

### Comparator model performance

**Fig 1:**
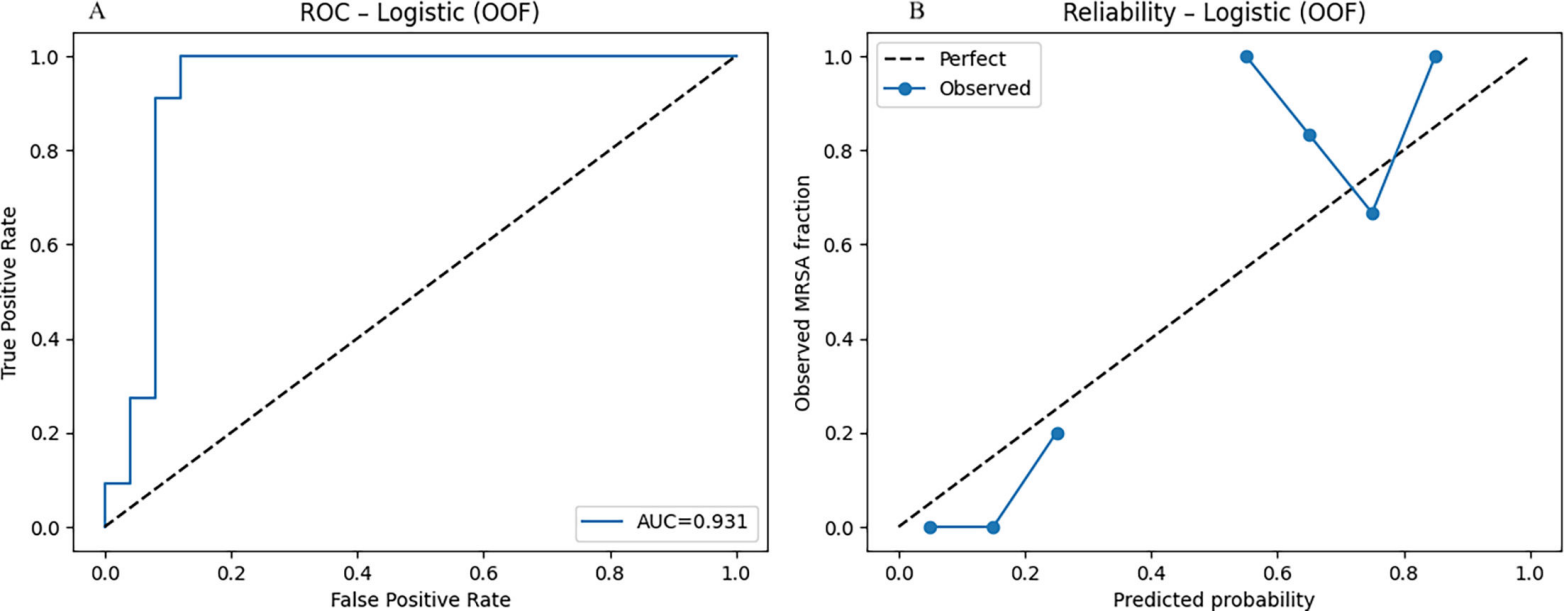
Logistic regression model’s discrimination and calibration. Receiver operating characteristic (ROC, panel **A**) and calibration (panel **B**) curves for logistic regression in the internal cohort (*n* = 93; 36 culture-confirmed *S. aureus*, including 11 MRSA). The model achieved an AUC of 0.931, accuracy of 91.7%, and a Brier score of 0.093, the lowest among all classifiers tested. ROC analysis confirmed strong discrimination, while calibration analysis showed probabilities were reasonably aligned with observed MRSA fractions, though slight underestimation occurred at intermediate probability levels.

To benchmark performance across different machine learning approaches, we evaluated four classifiers using the same ΔCt-derived features and fivefold cross-validation: calibrated logistic regression, linear support vector machine (SVM), random forest, and histGradientBoosting (Table S2). Logistic regression (Fig. 1A and B) achieved the best overall balance of discrimination and calibration, with an accuracy of 91.7%, an AUC of 0.931, and the lowest Brier score (0.093). The linear SVM performed similarly in terms of discrimination (AUC 0.927, accuracy 91.7%) but showed poorer probability calibration (Fig. S2A and B). Random forest performance was lower, with an AUC of 0.865 and an accuracy of 88.9% (Fig. S3A and B). HistGradientBoosting failed to generalize, with an AUC of 0.464 and accuracy of 69.4%, reflecting overfitting in this modest data set (Fig. S4A and B). Among the four models, logistic regression provided the most balanced combination of discrimination and calibration, supporting its selection as the primary analytic framework for attributing *mecA* in polymicrobial wound specimens.

### Logistic regression performance

In the 36 culture-confirmed *S. aureus* cases (Table 2), the logistic regression model achieved 91.7% overall accuracy (33/36). Sensitivity for MRSA was 90.9% (10/11), and specificity for MSSA was 92.0% (23/25). The cross-validated ROC AUC was 0.931, and the Brier score was 0.093 (Table 3). The confusion matrix contained 23 true negatives, 10 true positives, 2 false positives, and 1 false negative.

**TABLE 2:** Specimen provenance and PCR characteristics of 36 culture-positive *Staphylococcus aureus* cases^*a*^

Case	Culture_SA	PCR_SA	Ct_SA	PCR_*mecA*	Ct_*mecA*	Ct_CoNS	dCt_SA	dCt_CoNS	AST_SA	Fold	oof_prob	oof_pred	Result
3	Positive	Positive	19.15	Negative	NaN	33.29	NaN	NaN	MSSA	2	0.170304	MSSA	TN
6^*b*^	Positive	Negative	NaN	NaN	NaN	NaN	NaN	NaN	MRSA	4	0.273535	NaN	FN
10	Positive	Positive	28.90	Negative	NaN	31.93	NaN	NaN	MSSA	4	0.180230	MSSA	TN
12	Positive	Positive	19.18	Positive	20.24	22.37	1.06	−2.13	MSSA	1	0.694728	MRSA	FP
15	Positive	Positive	16.17	Positive	32.50	21.60	16.33	10.90	MSSA	5	0.110147	MSSA	TN
16	Positive	Positive	19.17	Positive	25.05	27.60	5.88	−2.55	MSSA	3	0.206905	MSSA	TN
17	Positive	Positive	17.14	Negative	NaN	NaN	NaN	NaN	MSSA	2	0.151834	MSSA	TN
19	Positive	Positive	15.34	Positive	14.57	33.96	−0.77	−19.39	MRSA	2	0.691481	MRSA	TP
22	Positive	Positive	17.14	Positive	28.18	28.43	11.04	−0.25	MSSA	3	0.077272	MSSA	TN
25	Positive	Positive	24.35	Positive	25.03	28.47	0.68	−3.44	MRSA	5	0.576307	MRSA	TP
27	Positive	Positive	18.80	Positive	18.02	24.00	−0.78	−5.98	MRSA	2	0.659835	MRSA	TP
28	Positive	Positive	18.70	Positive	33.77	27.50	15.07	6.27	MSSA	1	0.064823	MSSA	TN
36	Positive	Positive	19.02	Positive	31.16	30.80	12.14	0.36	MSSA	5	0.064932	MSSA	TN
39	Positive	Positive	17.02	Negative	NaN	28.00	NaN	NaN	MSSA	2	0.155345	MSSA	TN
41	Positive	Positive	20.02	Positive	30.51	33.94	10.49	−3.43	MSSA	1	0.118248	MSSA	TN
45	Positive	Positive	19.02	Positive	33.96	NaN	14.94	NaN	MSSA	3	0.095549	MSSA	TN
48	Positive	Positive	20.50	Positive	19.75	33.00	−0.75	−13.25	MRSA	1	0.808624	MRSA	TP
55	Positive	Positive	22.94	Positive	32.87	NaN	9.93	NaN	MSSA	4	0.126855	MSSA	TN
56	Positive	Positive	20.85	Positive	33.37	NaN	12.52	NaN	MSSA	1	0.092327	MSSA	TN
58	Positive	Positive	21.85	Positive	21.02	32.70	−0.83	−11.68	MSSA	1	0.786034	MRSA	FP
71	Positive	Positive	18.70	Positive	17.91	NaN	−0.79	NaN	MRSA	3	0.644986	MRSA	TP
75	Positive	Positive	18.62	Negative	NaN	NaN	NaN	NaN	MSSA	5	0.239502	MSSA	TN
79	Positive	Positive	21.79	Positive	24.95	28.17	3.16	−3.22	MSSA	4	0.285449	MSSA	TN
83	Positive	Positive	21.44	Positive	20.53	NaN	−0.91	NaN	MRSA	1	0.727802	MRSA	TP
84	Positive	Positive	22.40	Positive	21.11	31.32	−1.29	−10.21	MRSA	1	0.753935	MRSA	TP
86	Positive	Positive	24.70	Negative	NaN	NaN	NaN	NaN	MSSA	5	0.205249	MSSA	TN
88	Positive	Positive	26.40	Positive	25.12	NaN	−1.28	NaN	MRSA	4	0.542250	MRSA	TP
89	Positive	Positive	20.70	Positive	20.03	NaN	−0.67	NaN	MRSA	3	0.634048	MRSA	TP
92	Positive	Positive	27.00	Positive	33.49	NaN	6.49	NaN	MSSA	4	0.167130	MSSA	TN
93	Positive	Positive	30.10	Negative	NaN	NaN	NaN	NaN	MSSA	5	0.179034	MSSA	TN
95	Positive	Positive	22.15	Positive	33.58	34.14	11.43	−0.56	MSSA	2	0.066046	MSSA	TN
98	Positive	Positive	26.45	Positive	30.90	31.83	4.45	−0.93	MSSA	3	0.121615	MSSA	TN
100	Positive	Positive	31.30	Negative	NaN	NaN	NaN	NaN	MSSA	3	0.189011	MSSA	TN
103	Positive	Positive	16.90	Positive	15.89	20.42	−1.01	−4.53	MRSA	5	0.654238	MRSA	TP
104	Positive	Positive	30.70	Negative	NaN	NaN	NaN	NaN	MSSA	4	0.181366	MSSA	TN
107	Positive	Positive	16.12	Positive	25.37	25.89	9.25	−0.52	MSSA	2	0.100909	MSSA	TN

^*a*^ Ct_SA, cycle threshold for *S. aureus*; Ct_*mecA*, cycle threshold for *mecA*; Ct_CoNS, cycle threshold for coagulase-negative *staphylococci*; dCt_SA, Ct_mecA minus Ct_SA; dCt_CoNS, Ct_mecA minus Ct_CoNS; AST_SA, phenotypic susceptibility (MSSA vs MRSA); oof_prob, out-of-fold predicted probability; oof_pred, out-of-fold predicted label; result, classification outcome (TP, TN, FP, FN); MSSA, methicillin-sensitive *S. aureus*; MRSA, methicillin-resistant *S. aureus*; CoNS, coagulase-negative *staphylococci*; SA, *S. aureus*; NaN, not available.

^*b*^ In the culture-positive reference cohort, this case is treated as an FN for* S. aureus. *

**TABLE 3:** Logistic regression performance (36 culture-positive cases, OOF CV)^*a*^

Metric	Estimate	95% CI	Numerator/denominator
Accuracy	0.917	0.78–0.97	33/36
Sensitivity	0.909	0.62–0.98	10/11
Specificity	0.920	0.75–0.98	23/25
Brier score	0.093	–^*b*^	–

^*a*^ OOF cross-validation performance of the ΔCt-informed logistic regression in culture-positive *S. aureus* cases.

^*b*^ – indicates value not calculated, as Brier score is prevalence dependent.

**Fig 2:**
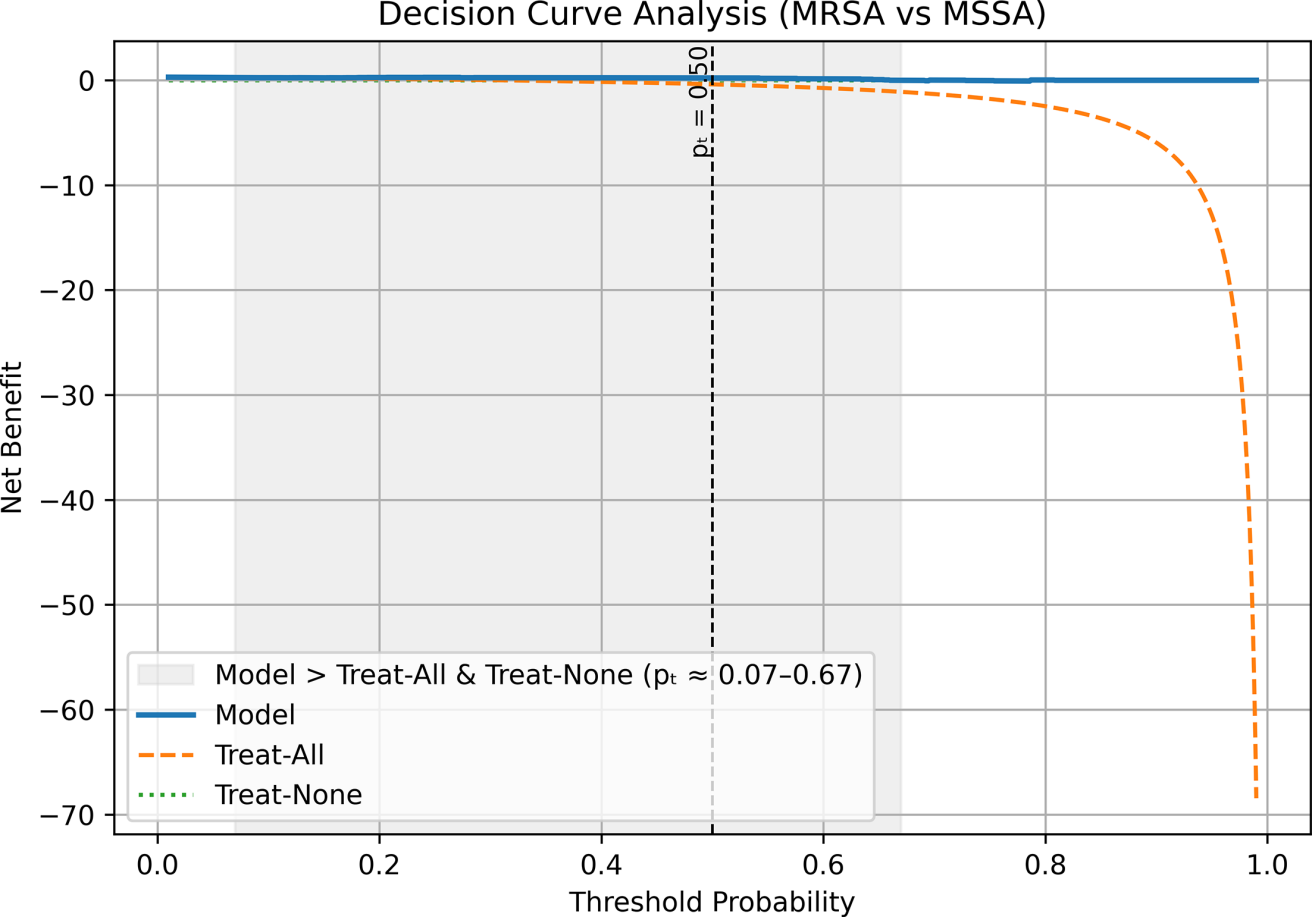
Decision curve analysis of the logistic regression model for MRSA versus MSSA. Net benefit is shown across threshold probabilities (*p*_*t*_) for the ΔCt-informed logistic regression model (solid line), a treat-all-as-MRSA strategy (dashed line), and a treat-none strategy (horizontal reference line at zero net benefit). The shaded region indicates the primary threshold interval over which the model provides higher net benefit than both default strategies (approximately *p*_*t*_ = 0.07–0.67), reflecting improved discrimination that reduces unnecessary MRSA-directed therapy while maintaining MRSA coverage. The vertical dashed line marks the prespecified reporting threshold (*p*_*t*_ = 0.50), at which the model achieved a net benefit of 0.22, whereas the treat-all strategy yielded a negative net benefit. Decision curve analysis evaluates clinical utility across ranges of threshold probabilities rather than defining a single statistical “significance point.”

We also compared the model with a prevalence-only null model and calculated the Brier skill score (BSS), also known as the index of prediction accuracy (IPA). The model’s Brier score (0.093) was substantially lower than the null score (0.212, MRSA prevalence = 30.6%), corresponding to a BSS/IPA of 0.56, or a 56% improvement over baseline. Together with the AUC of 0.931, these results indicate strong discrimination and reliable probability estimates. Decision curve analysis further showed that the model provided greater net benefit than default strategies of treating all or treating none. At the prespecified 50% cutoff for out-of-fold probabilities, the model achieved a net benefit of 0.222, while the treat-all approach (i.e., with anti-MRSA targeted antibiotics) produced a negative benefit (−0.389). These findings demonstrate that the model not only distinguishes MRSA from MSSA with high accuracy but also offers clear clinical value by reducing unnecessary MRSA therapy. Figure 2 shows the decision curve analysis across clinically relevant thresholds.

### Model calibration, robustness, and stability

**Fig 3:**
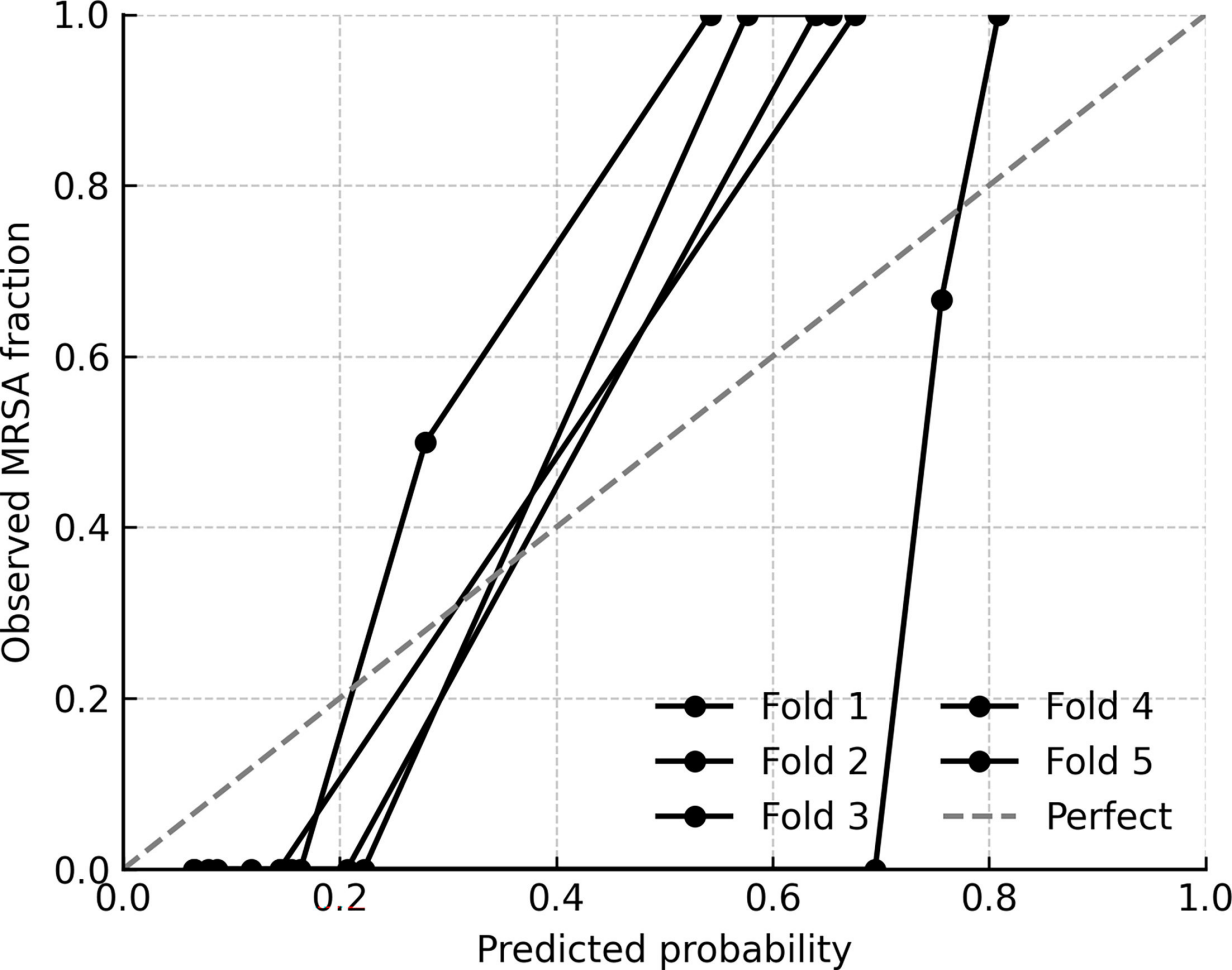
Calibration across five cross-validation folds. Calibration (reliability) curves are shown for each of the five cross-validation folds in the internal cohort. Curves deviated from the ideal 1:1 diagonal, particularly at mid-range predicted probabilities, where the model underestimated observed MRSA prevalence. Expected calibration errors ranged from 0.18 to 0.22 (mean ≈ 0.20), consistent with the modest number of MRSA cases per fold. Despite moderate miscalibration, overall discrimination remained strong.

**Fig 4:**
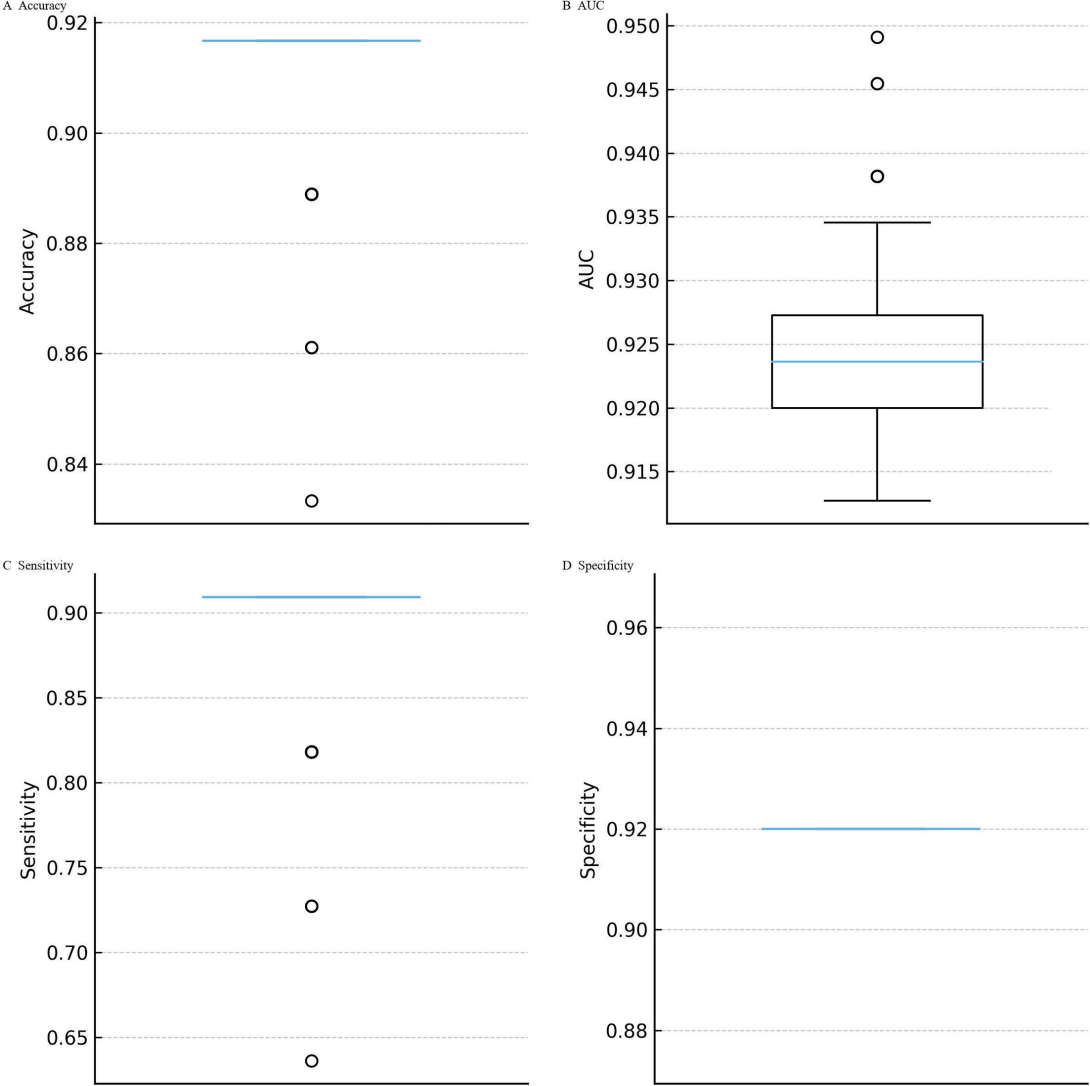
Stability of logistic regression performance across repeated cross-validation. (**A–D**) Distributions of accuracy, AUC, sensitivity, and specificity are shown across 100 iterations of stratified fivefold cross-validation. The mean accuracy was 0.911 (SD = 0.015; range 0.833–0.917), mean AUC 0.926 (SD = 0.007; range 0.913–0.949), sensitivity 0.890 (SD = 0.049; range 0.636–0.909), and specificity consistently 0.920 (SD ≈ 0). Narrow interquartile ranges confirmed reproducibility of accuracy, AUC, and sensitivity. Variability was driven almost entirely by sensitivity, reflecting the limited number of MRSA cases per fold.

**Fig 5:**
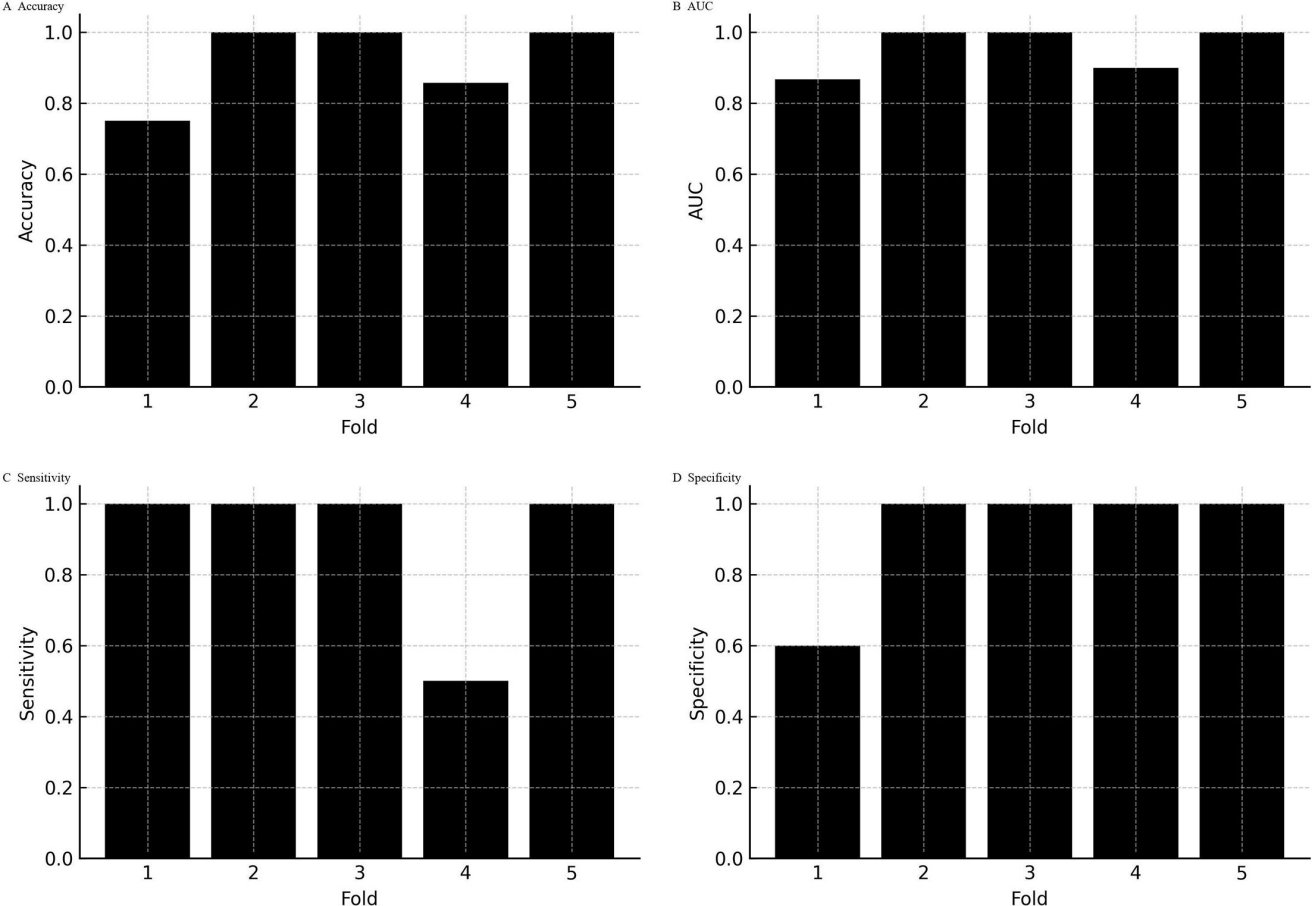
Per-fold performance within an exemplar split. Performance metrics (**A–D**) are displayed for each of the five folds in a representative cross-validation split. Specificity ranged from 0.60 to 1.00 (mean 0.92; per-fold SD = 0.179), highlighting within-split variability despite consistently high average discrimination.

**Fig 6:**
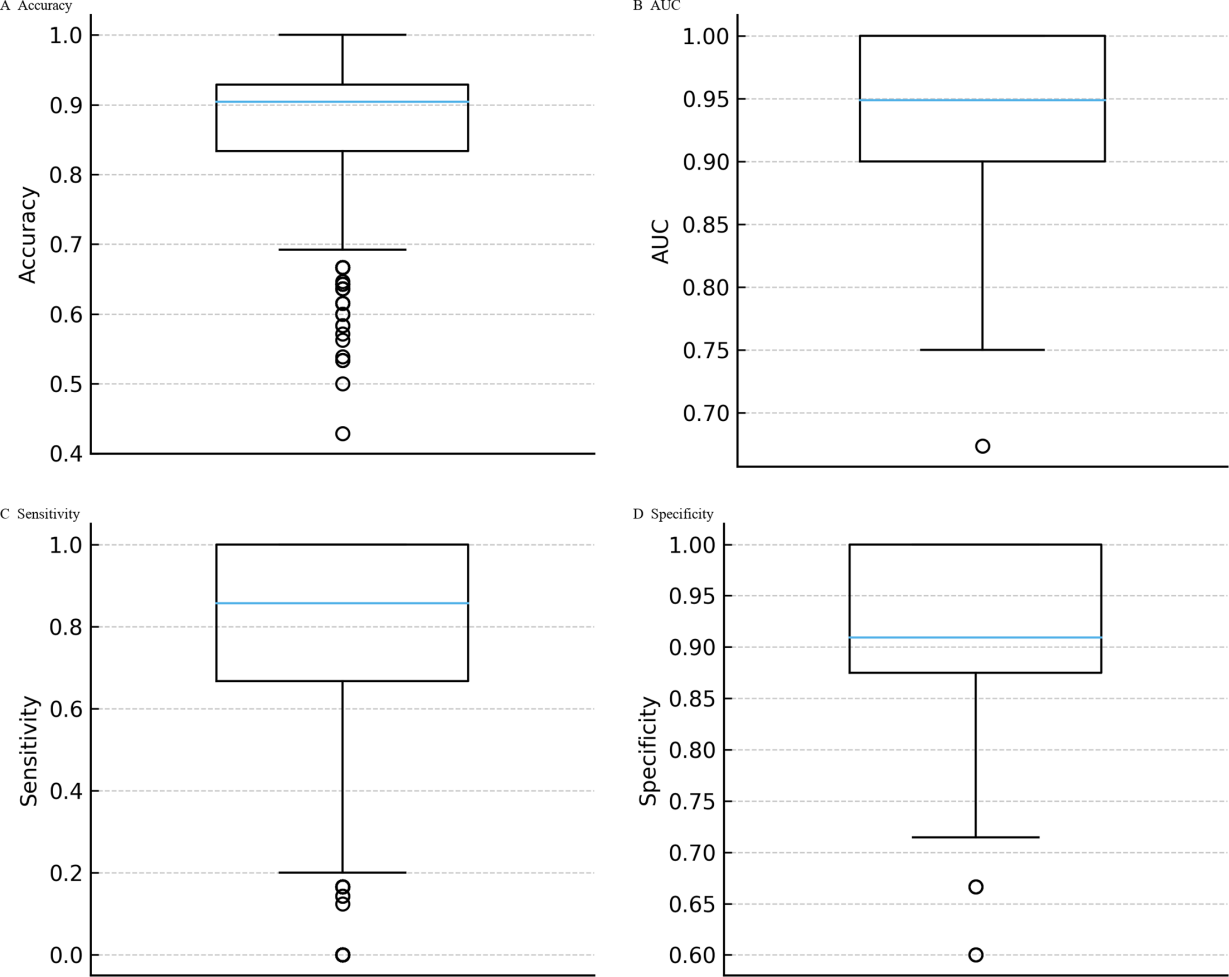
Bootstrap distributions of model performance. Nonparametric bootstrap resampling of the 36 culture-confirmed *S. aureus* cases (25 MSSA, 11 MRSA) was used to quantify uncertainty. Median estimates were 0.878 for accuracy (95% CI 0.64–1.00), 0.945 for AUC (0.83–1.00), 0.804 for sensitivity (0.17–1.00), and 0.921 for specificity (0.75–1.00) (**A–D**). These distributions confirm that observed performance metrics are robust to resampling and not artifacts of a particular partitioning scheme.

**Fig 7:**
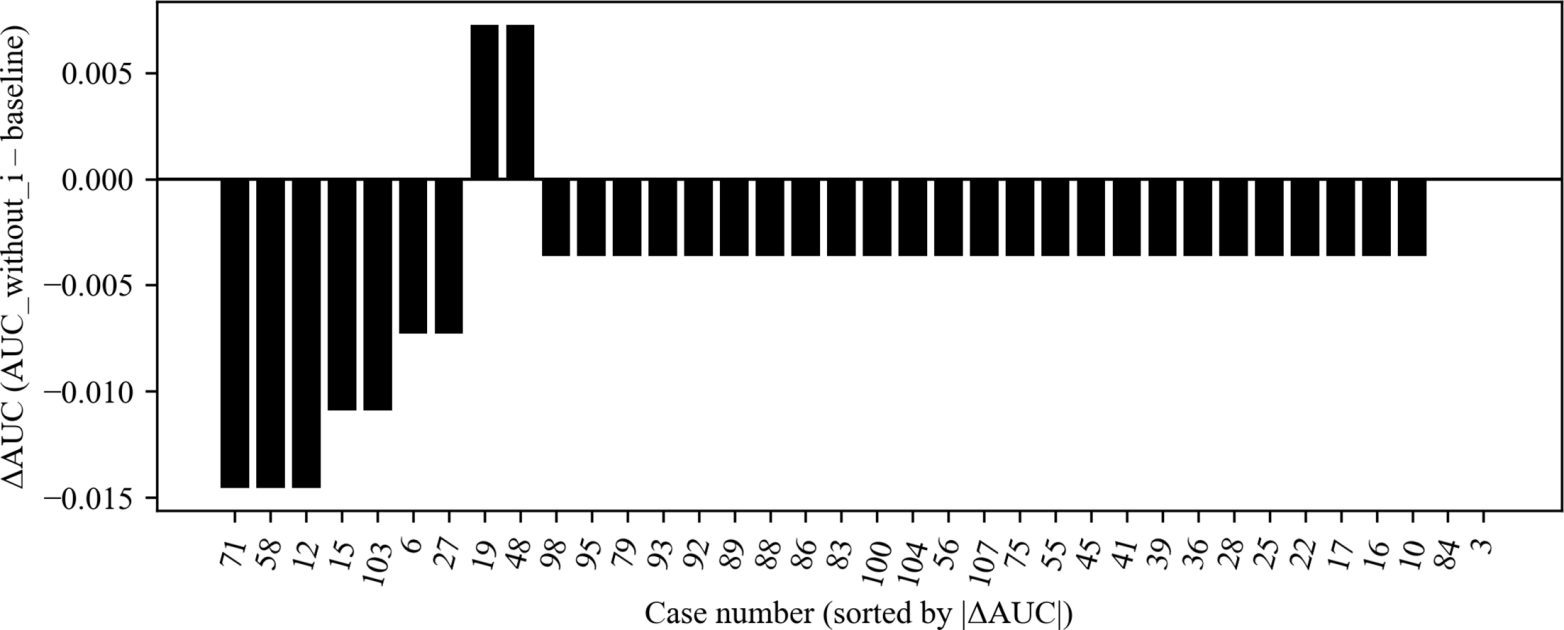
Jackknife validation of AUC estimates. Leave-one-out (jackknife) resampling of the 36 culture-confirmed *S. aureus* cases assessed the influence of individual specimens on AUC. Omitting any single case altered the AUC by no more than 0.015. Only five specimens produced absolute ΔAUC values >0.01, each corresponding to borderline ΔCt values near the classification threshold. The remaining 31 specimens had negligible impact (|ΔAUC| ≤ 0.007), confirming the robustness of the logistic regression model to individual case influence.

Beyond point estimates of sensitivity and specificity, it is essential to evaluate whether a diagnostic model produces probabilities that are reliable, stable across resampling schemes, and robust to individual case influence. Therefore, we examined calibration, repeated cross-validation, fold-level performance, bootstrap resampling, and jackknife influence (Fig. 3 to 7).

Across the five cross-validation folds, the calibration curves deviated from the ideal 1:1 line, particularly at mid-range predicted probabilities where the model tended to underestimate the observed MRSA fraction. Expected calibration errors ranged from 0.18 to 0.22 (mean ≈ 0.20), reflecting moderate miscalibration consistent with the limited number of MRSA cases per fold (Fig. 3). Despite this, classification metrics remained strong. Repeated 100 × 5-fold cross-validation demonstrated that the logistic regression model’s performance was stable across resampling runs. The mean accuracy was 0.911 (SD 0.015), with values ranging from 0.833 to 0.917. The mean AUC was 0.926 (SD 0.007), spanning 0.913 to 0.949. Sensitivity averaged 0.890 (SD 0.049), varying between 0.636 and 0.909, while specificity was consistently 0.920 across all folds (SD ≈0). Interquartile ranges were narrow for accuracy, AUC, and sensitivity, underscoring reproducibility of the model’s discrimination. Variability was almost entirely driven by sensitivity, reflecting the limited number of MRSA cases within each fold (Fig. 4). At the per-fold level within an exemplar split, specificity ranged 0.60–1.00 (mean 0.92; per-fold SD = 0.179), illustrating within-split variability (Fig. 5). To summarize uncertainty, while avoiding aggregation ambiguity, nonparametric bootstrap over the 36 culture-confirmed cases gave specificity 0.921 (95% CI 0.75–1.00), sensitivity 0.804 (0.17–1.00), accuracy 0.878 (0.64–1.00), and AUC 0.945 (0.83–1.00) (Fig. 6). These distributions confirm that the observed performance metrics are not artifacts of a single partitioning scheme. Finally, jackknife analysis showed that omitting any single case changed the AUC by no more than 0.015. Only 5 of the 36 specimens produced absolute ΔAUC values exceeding 0.01; these were cases with probabilities near the decision threshold, highlighting how borderline ΔCt values can slightly influence performance. The other 31 observations had negligible impact (|ΔAUC| ≤ 0.007), underscoring the robustness of the model to individual cases (Fig. 7).

Collectively, these analyses show that the logistic regression model generalizes well across resampling schemes and is robust to individual cases. Calibration analysis demonstrated moderate deviation between predicted MRSA probabilities and observed MRSA frequencies, particularly in the mid-probability range. This degree of miscalibration is expected in modest data sets with a limited number of MRSA events per fold and occurred despite strong discrimination (AUC ≈ 0.93). Calibration (reliability) curves are provided in the supplemental material.

Notably, while the number of MRSA cases was limited, the number of MSSA specimens was substantially larger, and the model demonstrated consistently high specificity across resampling schemes, indicating reliable discrimination of methicillin-susceptible *Staphylococcus aureus* despite class imbalance. Because probabilities generated by this framework are intended to support tiered interpretation rather than fixed categorical reporting, laboratories implementing this approach may consider local recalibration based on prevalence and workflow priorities.

### Application to the full cohort

The calibrated model with *S. aureus* gating was applied across all 93 wound specimens (Table S3). Among the 57 specimens that were culture-negative for *Staphylococcus aureus*, 55 were negative for *S. aureus* by PCR and were reported as “No MSSA/MRSA.” Two specimens were PCR-positive for *S. aureus* but culture-negative and were treated as PCR-culture discordant; these cases were excluded from MRSA/MSSA attribution accuracy denominators.

Among the 36 specimens that were culture-positive for *S. aureus*, the model produced 23 MSSA calls, 12 MRSA calls (including 2 discordant relative to phenotypic culture and susceptibility testing), while one specimen was gated as “No MSSA/MRSA” because PCR was negative for both *S. aureus* and *mecA*. This distribution aligns with the validated performance metrics observed in the AST-defined reference cohort (sensitivity 90.9% and specificity 92.0%), while extending the same biologic plausibility rules to the full data set. Overall, model outputs across all 93 specimens consisted of 55 “No MSSA/MRSA,” 26 MSSA, and 12 MRSA classifications.

Under the biologic gating rule, MRSA/MSSA attribution is performed only when *S. aureus* is detected by PCR; when *S. aureus* PCR is negative, model output is reported as “No MSSA/MRSA,” reflecting that *mecA* attribution is not biologically meaningful in the absence of a detected *S. aureus* signal. For this specimen, phenotypic culture and AST nevertheless identified *S. aureus* and reported methicillin susceptibility by standard-of-care testing.

### External validation

In the independent external cohort (*n* = 47), culture identified 13 specimens positive for *Staphylococcus aureus*. The PCR panel detected *S. aureus* in 12 of these, yielding a sensitivity of 92.3% (12/13) compared with culture, corresponding to one culture-confirmed MRSA specimen that was PCR-negative for *S. aureus* (false negative for species detection). Among the 34 culture-negative specimens, PCR results were negative in 33 and positive in 1 (Case #218), giving a specificity of 97.1% (33/34) and representing one false-positive PCR result for *S. aureus* detection (Table 4). The combined “Internal + External” row in Table 4 summarizes overall descriptive performance across all evaluable *S. aureus* PCR-positive specimens from both cohorts and is provided for reference only; primary performance estimates are reported separately for the internal and external cohorts.

**TABLE 4:** Performance of PCR panel for *S. aureus* detection and MRSA attribution in internal and external cohorts^*a*^

Task	Cohort	*n*	Sensitivity (95% CI)	Specificity (95% CI)	Accuracy (95% CI)	PPV	NPV
*S. aureus* detection vs culture	Internal	93	97.2% (85.8–99.5)	94.7% (85.6–98.2)	95.7% (89.5–98.3)	92.1%	98.2%
	External	47	92.3% (66.7–98.6)	97.1% (85.1–99.5)	95.7% (85.8–98.8)	92.3%	97.1%
MRSA attribution (SA+ only)	Internal	36	90.9% (62.3–98.4)	92.0% (75.0–97.8)	91.7% (78.2–97.1)	83.3%	95.8%
	External	12	100% (51.0–100)	87.5% (52.9–97.8)	91.7% (64.6–98.5)	80.0%	100%
	Internal + External	48	93.3% (70.2–98.8)	90.9% (76.4–96.9)	91.7% (80.4–96.7)	82.4%	96.8%

^*a*^ *S. aureus* detection was evaluated against culture as the reference standard. MRSA attribution was evaluated only among *S. aureus* PCR-positive specimens after applying the biologic gating rule. 95% confidence intervals for sensitivity, specificity, and accuracy were calculated using the Wilson method. PPV and NPV are reported as point estimates only because they are prevalence-dependent.

For methicillin resistance attribution, the biologic gating rule was applied so that only *S. aureus* PCR-positive specimens were classified as MRSA or MSSA. Within this evaluable subset (*n* = 12), culture identified 4 MRSA and 8 MSSA cases. The calibrated logistic regression model correctly classified all MRSA cases, corresponding to a sensitivity of 100% for MRSA detection. Among MSSA cases, one was misclassified as MRSA, yielding a specificity of 87.5%. The overall accuracy for MRSA/MSSA classification in *S. aureus*-positive specimens was 91.7%, with positive and negative predictive values of 80% and 100%, respectively (Table S4).

## DISCUSSION

Polymicrobial wound specimens frequently contain *S. aureus* alongside CoNS, many of which carry the *mecA* gene. This overlap complicates interpretation, because *mecA* co-detection with *S. aureus* by PCR does not necessarily imply MRSA. Misattribution of *mecA* to *S. aureus* may result in unnecessary use of glycopeptides or oxazolidinones rather than the recommended β-lactams for MSSA, potentially exposing patients to greater toxicity, higher cost, and more complicated treatment courses (1, 2). In polymicrobial wound infections, particularly in chronic and podiatric cases, therapeutic options are sometimes constrained by polymicrobial burden, patient comorbidities, and antimicrobial toxicity. In this context, a conservative interpretation of any *mecA* detection as MRSA may unnecessarily restrict therapy options, motivating the need for a more principled interpretive framework.

To address this challenge, we incorporated ΔCt features, defined as the Ct difference between *mecA* and staphylococcal targets such as *S. aureus* or CoNS, into a calibrated, class-weighted logistic regression model, combined with a biologic gate that suppressed MRSA/MSSA classification when *S. aureus* PCR was negative. In the 36 culture-confirmed *S. aureus* cases, our logistic regression model achieved 91.7% accuracy, with sensitivity for MRSA of 90.9% and specificity for MSSA of 92.0%. The model showed strong discrimination (AUC = 0.931) and a low Brier score (0.093), indicating overall good calibration. However, because the Brier score is prevalence-dependent and does not directly capture clinical value (13), we also compared the model with a prevalence-only null model. This yielded a Brier skill score of 0.56, reflecting a 56% improvement in probabilistic accuracy over baseline. To further evaluate clinical usefulness, we applied decision curve analysis. At the prespecified 50% probability cutoff, the model achieved a net benefit of 0.222, while the treat-all approach, equivalent to treating all *S. aureus* positive cases as MRSA, resulted in a negative net benefit (−0.389). These findings demonstrate that the model distinguishes MRSA from MSSA accurately and supports more selective MRSA attribution compared with a treat-all strategy.

Our approach builds on prior ΔCt-based strategies used in commercial assays (6). For example, the MRSA/SA ELITe MGB kit interprets ΔCt <2 cycles between *mecA* and an *S. aureus* target as MRSA, with 100% concordance in a study of 82 nasal isolates, whereas the Xpert MRSA/SA assay showed lower concordance (76.8%) (1, 2, 6). These assays are optimized for MRSA/*Staphylococcus aureus* screening or isolate-based testing and incorporate assay-specific targets or interpretation rules to support MRSA identification in those contexts. In contrast, many panel-based wound PCR tests report organism and resistance gene targets separately without direct genomic linkage, which can complicate *mecA* attribution in polymicrobial wound specimens where coagulase-negative staphylococci frequently harbor mecA.

Our model achieved comparable accuracy of 91.7% in both the external and internal cohorts, while providing probabilistic outputs and explicit biologic gating. Unlike fixed cutoffs, logistic regression incorporates multiple features (Ct, ΔCt, detection flags) and generates calibrated probabilities that can be adjusted to clinical settings.

Selective broth enrichment combined with *nuc–mecA* PCR has also been proposed, achieving 93.5% sensitivity and 88.6% specificity in 1,250 samples (7). Our sensitivity (90.9%) and specificity (92.0%) fall within this range, but our method operates directly on wound specimens that are often polymicrobial without enrichment. Third-generation droplet digital PCR assays have demonstrated excellent performance (>96% sensitivity, >91% specificity) in nasal swabs (14), but require specialized instrumentation. Our framework achieved comparable accuracy in wound specimens using probe-based real-time PCR assays, while incorporating the linkage probability to CoNS. Similarly, rapid multiplex PCR (15) and MALDI-TOF combined with machine learning (16) show the breadth of approaches for MRSA detection, yet differ in instrumentation requirements and scope. Our ΔCt-informed logistic regression complements these efforts by focusing on direct clinical wound specimens and explicitly linking *mecA* attribution to biologic plausibility. By embedding ΔCt features within a machine-learning framework and calibrating the resulting probabilities, this approach is designed to improve *mecA* attribution to *Staphylococcus aureus* in polymicrobial settings while behaving predictably in biologically complex or ambiguous scenarios that challenge both molecular and phenotypic methods. In specimens containing heterogeneous *Staphylococcus aureus* populations, including mixtures of MRSA and MSSA populations, ΔCt patterns may yield intermediate predicted probabilities. Such outputs are biologically plausible and are expected to cluster near the decision threshold rather than producing extreme probabilities. Importantly, similar ambiguity can arise in phenotypic AST depending on colony selection in mixed populations. The probabilistic outputs of this framework are therefore intended to surface underlying biological uncertainty rather than obscure it.

It should be noted that a key limitation of this framework is that it is designed specifically to attribute *mecA*-mediated resistance and therefore does not detect borderline oxacillin-resistant *Staphylococcus aureus* phenotypes, which are typically *mecA*-negative and arise from alternative resistance mechanisms. In such cases, the model would not infer MRSA despite reduced oxacillin susceptibility, underscoring the continued necessity of phenotypic AST for detecting non–mecA-mediated resistance and reinforcing that this framework is intended to complement, rather than replace, conventional susceptibility testing.

In our wound cohort, 25 of 36 culture-positive *S. aureus* cases were methicillin-sensitive (MSSA; 69%), underscoring the importance of distinguishing between *S. aureus* and CoNS as carriers of mecA. A similar distribution was observed in the external validation set, where 8 of 12 *S. aureus*-positive cases were MSSA (67%) and 4 were MRSA (33%). By combining ΔCt quantification with biologic gating, our framework directly addresses this limitation and reduces inappropriate MRSA attribution. The use of calibrated probabilities further provides transparency and auditability, features that may enhance clinician confidence in molecular resistance reporting. While stand-alone MRSA PCR kits exist and are valuable for screening, they are not designed for polymicrobial wound specimen testing. Our framework is intended to enhance the panel-based TaqMan assays already offered as LDTs in CAP/CLIA labs, improving mecA attribution within multiplex workflows without requiring additional testing.

As a secondary finding, PCR demonstrated high accuracy for *S. aureus* detection in the external validation cohort (*n* = 47), with 92.3% sensitivity, 97.1% specificity, and 95.7% overall accuracy, closely matching the performance observed in our prior evaluation of the internal cohort.

Because specimens were collected using paired swabs rather than replicate testing of the same material, some discordance between PCR and culture is expected and reflects known differences in analytical sensitivity and sampling variability. The objective of this study was not to adjudicate PCR-culture discordance or establish a composite reference standard but rather to evaluate a ΔCt-informed framework for attributing *mecA* within PCR-positive wound specimens using culture/AST as the reference standard where *S. aureus* was recovered.

Culture-negative/PCR-positive *S. aureus* cases were therefore handled transparently as discordant relative to the reference standard and were excluded from MRSA/MSSA attribution accuracy denominators. Prospective clinical implementation may benefit from reflex or adjudication strategies (e.g., repeat culture, repeat molecular testing, or targeted confirmation), but retrospective adjudication was not attempted in this quality-assurance data set.

### Limitations and future directions

The main limitation of this study was the modest sample size, particularly the small number of MRSA cases, which contributed to variability in sensitivity estimates and limited generalizability. Culture served as the reference standard but may misclassify borderline or mixed infections. Although the underlying classification performance was strong, as shown by AUC values, Brier score, and positive net benefit, the reliability curves show moderate miscalibration. Larger external cohorts are needed to confirm performance, refine calibration, and quantify inter-laboratory variability.

### Conclusions

Our ΔCt-informed, *S. aureus*-gated logistic regression demonstrated accurate and interpretable *mecA* attribution in wound specimens, with balanced sensitivity and specificity. By embedding biologic plausibility and PCR kinetics, the model supports more selective MRSA interpretation in polymicrobial settings. Importantly, decision curve analysis demonstrated that this approach yielded a positive net benefit compared with treating all *S. aureus* cases as MRSA, indicating that the model enhances both clinical decision-making and statistical accuracy. PCR also maintained high accuracy for *S. aureus* detection in an external validation set, underscoring the robustness of the molecular workflow. Although our findings are encouraging, the number of MRSA cases was modest, and larger, more diverse cohorts will be needed to validate calibration, refine cutoff rules, and confirm clinical utility. If validated, this framework could be integrated into standard probe-based wound PCR panel workflows to enhance *mecA* interpretation within multi-target or panel-based PCR testing without additional laboratory burden.

## Data Availability

All analysis artifacts, including the case‑level provenance tables with out‑of‑fold probabilities and fold assignments, the 93‑case calibrated model outputs with *S. aureus* PCR gating applied, and the reproducible Python script used for analysis (mrsa_audit_pipeline.py), are available from the authors upon reasonable request in accordance with institutional data‑sharing policies.

## SUPPLEMENTAL MATERIAL

Supplemental figures spectrum.03230-25-s0001.pdf Figure S1 to S4.

Supplemental material spectrum.03230-25-s0002.docx Python codes.

Table S1 spectrum.03230-25-s0003.xlsx Master dataset of 93 cases.

Table S2 spectrum.03230-25-s0004.xlsx Model comparison across classifiers.

Table S3 spectrum.03230-25-s0005.xlsx Final classification of 93 wound specimens after *S. aureus* PCR gating.

Table S4 spectrum.03230-25-s0006.xlsx External validation.

ASM does not own the copyrights to Supplemental Material that may be linked to, or accessed through, an article. The authors have granted ASM a non-exclusive, world-wide license to publish the Supplemental Material files. Please contact the corresponding author directly for reuse.
