# Supplementary material for: ΔCt-informed, calibrated logistic regression accurately attributes mecA in Staphylococcus aureus-positive wound specimens
Source: Microbiol Spectr. 2026 Mar 31;14(5):e03230-25. doi: 10.1128/spectrum.03230-25 (PMC13141963; doi:10.1128/spectrum.03230-25)
Supplement: Supplemental figures — Figure S1 to S4. [file spectrum.03230-25-s0001.pdf]

Supplementary Figures

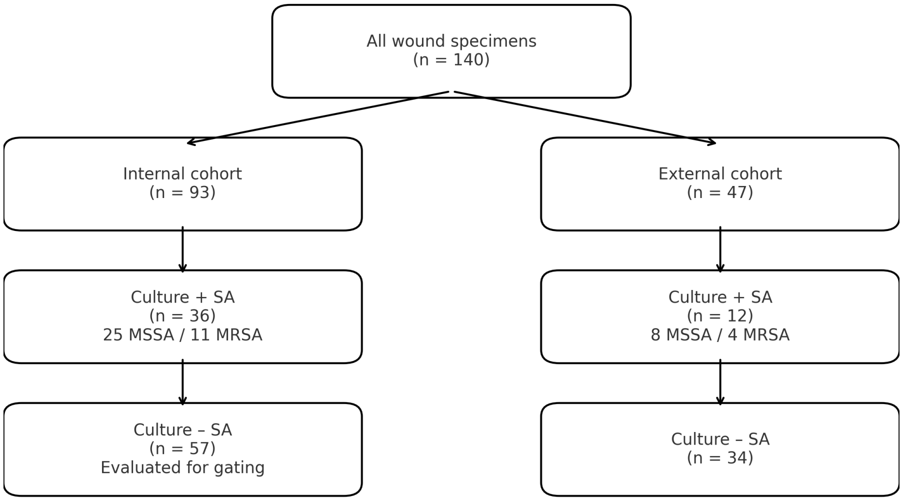

Supplementary Figure S1. STARD flow diagram for specimen inclusion and classification. From 140 total wound specimens, 93 were allocated to the internal training cohort and 47 to an external validation cohort. Within the internal cohort, 36 specimens were culture-positive for *S. aureus* (25 MSSA, 11 MRSA) and 57 were culture-negative, forming the dataset for model training and cross-validation. The external cohort comprised 12 culture-positive *S. aureus* specimens (8 MSSA, 4 MRSA) and 34 culture-negative specimens, none of which were exposed to model training. This independent cohort was used exclusively for assessing model generalizability.

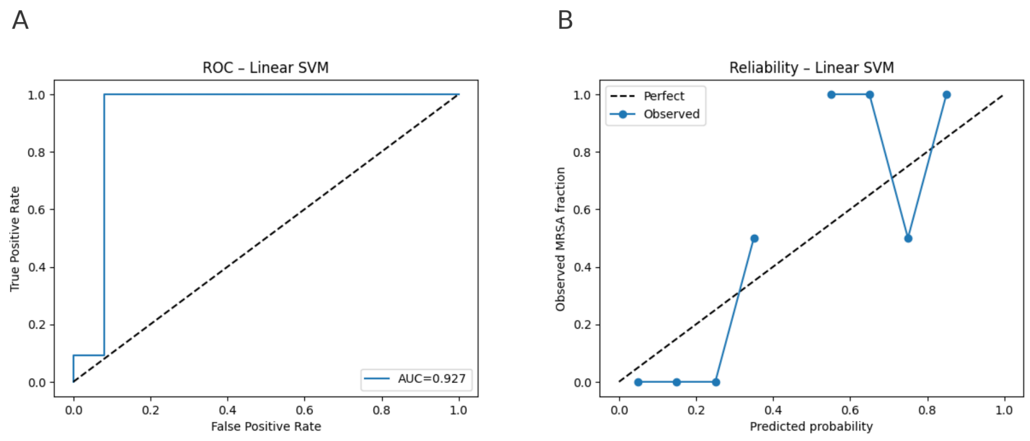

Figure S2. Performance of linear support vector machine (SVM). The linear SVM classifier trained on the internal cohort (n = 93) achieved an accuracy of 91.7%, an AUC of 0.927, and a Brier score of 0.105. The confusion matrix showed 23 true negatives, 2 false positives, 10 true positives, and 1 false negative, corresponding to a sensitivity of 0.91 and specificity of 0.92. The ROC curve (panel A) demonstrates strong discrimination. The calibration plot (panel B) compares predicted MRSA probabilities to observed outcomes across bins: each point represents a group of specimens with similar predicted risk, with the x-axis showing mean predicted probability and the y-axis the true fraction of MRSA. The SVM tended to overestimate probabilities in the mid-range while remaining well aligned at lower probabilities (The curve pulls away from the diagonal at mid-range predicted probabilities).

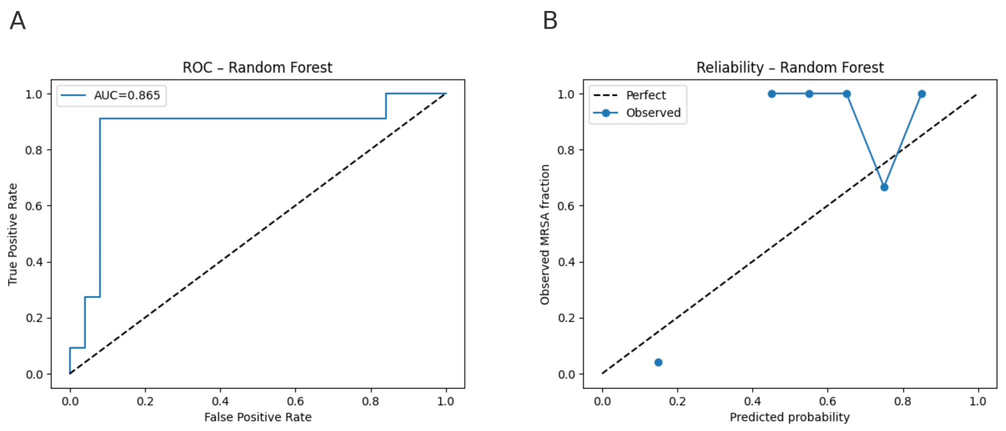

Figure S3. Performance of random forest classifier. The random forest model trained on the internal cohort achieved an accuracy of 88.9%, an AUC of 0.865, and a Brier score of 0.099. The confusion matrix indicated 23 true negatives, 2 false positives, 9 true positives, and 2 false negatives, corresponding to a sensitivity of 0.82 and specificity of 0.92. The ROC curve (panel A) shows moderate discrimination. The calibration plot (panel B) illustrates that predicted probabilities were less stable, with some bins substantially overestimating MRSA prevalence and others underestimating it, reflecting variability in reliability when probabilities were translated into risk estimates.

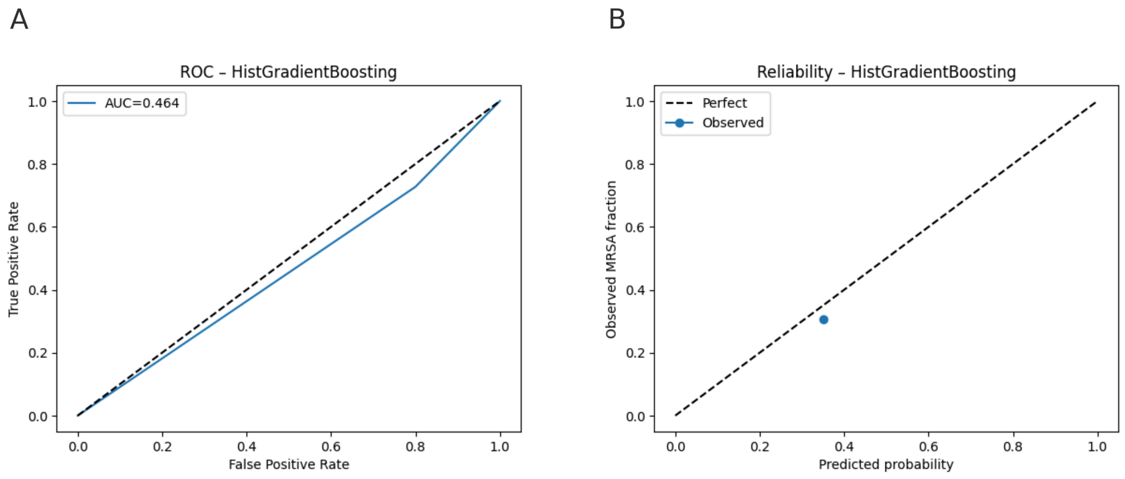

Figure S4. Performance of histogram-based gradient boosting. The HistGradientBoosting classifier performed poorly, with an accuracy of 69.4%, an AUC of 0.464, and a Brier score of 0.213. The confusion matrix showed 25 true negatives, 0 false

positives, 0 true positives, and 11 false negatives, yielding 0% sensitivity and 100% specificity. The ROC curve (panel A) indicates no meaningful discrimination. The calibration plot (panel B) shows that predicted probabilities clustered in the mid-range but failed to align with observed MRSA fractions; each point again represents a probability bin, but here none contained true MRSA cases, resulting in flat miscalibration.
