# Supplementary material for: ΔCt-informed, calibrated logistic regression accurately attributes mecA in Staphylococcus aureus-positive wound specimens
Source: Microbiol Spectr. 2026 Mar 31;14(5):e03230-25. doi: 10.1128/spectrum.03230-25 (PMC13141963; doi:10.1128/spectrum.03230-25)
Supplement: Supplemental material — Python codes. [file spectrum.03230-25-s0002.docx]

Supplementary: Python Code Index

# MRSA common.py

"""

mrsa_common.py

Shared utilities for MRSA vs MSSA (S. aureus phenotype) modeling.

- Loads: Master data_MRSA.xlsx (sheet "Table")

- ID: Case_No

- Features: Ct, ΔCt, |ΔCt|, logistic closeness for SA and CoNS, plus binary flags

- CV: 5-fold StratifiedKFold, probability calibration (Platt/sigmoid)

"""

from __future__ import annotations

from pathlib import Path

from typing import Tuple, List, Dict

import numpy as np

import pandas as pd

import matplotlib.pyplot as plt

from sklearn.model_selection import StratifiedKFold

from sklearn.calibration import CalibratedClassifierCV

from sklearn.metrics import (

accuracy_score,

roc_auc_score,

brier_score_loss,

confusion_matrix,

)

# ---------------------------------------------------------------------

# Configuration

# ---------------------------------------------------------------------

DATA_FILE = "Master data_MRSA.xlsx"

SHEET = "Table"

# For reference (cutoff already applied upstream to Positive/Negative calls)

CT_POS_CUTOFF = 34.0

# Canonical column mapping from the Excel to internal names

COLMAP: Dict[str, str] = {

"Case No": "Case_No",

"Culture S. aureus": "Culture_SA",

"PCR S. aureus": "PCR_SA_call",

"PCR S. aureus.1": "Ct_SA",

"PCR mecA Detected": "PCR_mecA_call",

"PCR mecA Ct": "Ct_mecA",

"coagulase-negative staphylococci Ct": "Ct_CoNS",

"Staphylococcus lugdunensis Ct": "Ct_SL", # optional; not used in FEATURES

"MRSA/MSSA (culture/AST)": "AST_SA_label",

}

# Unified feature set used by all models (Ct_SL removed to silence warnings)

FEATURES: List[str] = [

"Ct_mecA", "Ct_SA", "Ct_CoNS",

"dCt_SA", "adCt_SA", "P_SA",

"dCt_CoNS", "adCt_CoNS", "P_CoNS",

"PCR_SA_pos", "mecA_pos",

]

TARGET = "AST_SA_label"

ID_COL = "Case_No"

# ---------------------------------------------------------------------

# Helpers

# ---------------------------------------------------------------------

def _pos(x) -> "pd.Int64Dtype":

"""Map Positive/Negative strings (and common variants) to {1,0} (nullable Int)."""

if isinstance(x, str):

s = x.strip().lower()

if s in ("positive", "pos", "yes", "true", "1"):

return 1

if s in ("negative", "neg", "no", "false", "0"):

return 0

return pd.NA

def _closeness(delta_ct: float) -> float:

"""Logistic closeness transform: P(ΔCt) = 1 / (1 + e^{2(|ΔCt|-2)})."""

if pd.isna(delta_ct):

return np.nan

return 1.0 / (1.0 + np.exp(2.0 * (abs(delta_ct) - 2.0)))

def _make_calibrator(estimator, method: str = "sigmoid", cv: int = 3):

"""Create CalibratedClassifierCV compatible with both new (estimator=) and old (base_estimator=) sklearn."""

try:

# Newer sklearn (>=1.4-ish)

return CalibratedClassifierCV(estimator=estimator, method=method, cv=cv)

except TypeError:

# Older sklearn (<=1.3-ish)

return CalibratedClassifierCV(base_estimator=estimator, method=method, cv=cv)

# ---------------------------------------------------------------------

# IO + Feature engineering

# ---------------------------------------------------------------------

def load_master_mrsa(path: Path | str = DATA_FILE, sheet: str = SHEET) -> pd.DataFrame:

"""

Load 'Master data_MRSA.xlsx' and engineer features.

Returns a DataFrame with:

- Canonical columns (Case_No, Culture_SA, PCR_SA_call, Ct_* etc.)

- Binary flags: PCR_SA_pos, mecA_pos, Culture_SA_pos

- ΔCt and |ΔCt| for SA and CoNS (only when organism Ct present)

- Logistic closeness P_SA, P_CoNS (guarded against 'no signal')

- is_ground_truth (Culture_SA_pos == 1 and AST_SA_label not null)

"""

df = pd.read_excel(path, sheet_name=sheet)

# Rename to canonical internal names (ignore missing)

df = df.rename(columns={k: v for k, v in COLMAP.items() if k in df.columns})

# Coerce Ct numeric (tolerate missing)

for c in ("Ct_SA", "Ct_mecA", "Ct_CoNS", "Ct_SL"):

if c in df.columns:

df[c] = pd.to_numeric(df[c], errors="coerce")

# Binary flags from call columns

df["PCR_SA_pos"] = df["PCR_SA_call"].apply(_pos).astype("Int64")

df["mecA_pos"] = df["PCR_mecA_call"].apply(_pos).astype("Int64")

df["Culture_SA_pos"] = df["Culture_SA"].apply(_pos).astype("Int64")

# Detection flags based on Ct presence (no Ct == no signal)

df["CoNS_pos"] = df["Ct_CoNS"].notna().astype("Int64")

SA_ct_present = df["Ct_SA"].notna()

mecA_ct_present = df["Ct_mecA"].notna()

# ΔCt features (compute only when organism Ct is present)

# SA branch

df["dCt_SA"] = np.where(SA_ct_present & mecA_ct_present, df["Ct_mecA"] - df["Ct_SA"], np.nan)

df["adCt_SA"] = df["dCt_SA"].abs()

# CoNS branch

df["dCt_CoNS"] = np.where(df["CoNS_pos"].eq(1) & mecA_ct_present, df["Ct_mecA"] - df["Ct_CoNS"], np.nan)

df["adCt_CoNS"] = df["dCt_CoNS"].abs()

# Logistic closeness scores:

# - For SA: only when SA PCR is positive AND both Cts present; else 0 (no SA linkage evidence)

df["P_SA"] = np.where(

(df["PCR_SA_pos"].eq(1)) & SA_ct_present & mecA_ct_present,

df["dCt_SA"].apply(_closeness),

0.0

)

# - For CoNS: only when CoNS Ct present; else 0 (no CoNS linkage evidence)

df["P_CoNS"] = np.where(

df["CoNS_pos"].eq(1) & mecA_ct_present,

df["dCt_CoNS"].apply(_closeness),

0.0

)

# Ground-truth mask: culture-positive S. aureus with AST label present

df["is_ground_truth"] = (df["Culture_SA_pos"] == 1) & (df["AST_SA_label"].notna())

return df

def get_sa_dataset(df: pd.DataFrame) -> Tuple[pd.DataFrame, pd.Series, pd.Series]:

"""

Select ground-truth rows and return features X, labels y (MRSA=1/MSSA=0), ids (Case_No).

"""

G = df[df["is_ground_truth"]].copy()

X = G[FEATURES]

y = (G[TARGET] == "MRSA").astype(int)

ids = G[ID_COL].astype(str)

return X, y, ids

# ---------------------------------------------------------------------

# Cross-validated calibrated evaluation

# ---------------------------------------------------------------------

def cv_calibrated_eval(estimator, X: pd.DataFrame, y: pd.Series,

cv_splits: int = 5, seed: int = 42) -> dict:

"""

5-fold CV with Platt (sigmoid) calibration.

Returns dict with OOF probs/preds and metrics: acc, auc, brier, cm.

"""

skf = StratifiedKFold(n_splits=cv_splits, shuffle=True, random_state=seed)

oof_prob = np.zeros(len(y), dtype=float)

oof_pred = np.zeros(len(y), dtype=int)

for tr, te in skf.split(X, y):

X_tr, X_te = X.iloc[tr], X.iloc[te]

y_tr, y_te = y.iloc[tr], y.iloc[te]

# Calibrate on training fold (inner CV=3) and predict held-out fold

cal = _make_calibrator(estimator, method="sigmoid", cv=3)

cal.fit(X_tr, y_tr)

p = cal.predict_proba(X_te)[:, 1]

oof_prob[te] = p

oof_pred[te] = (p >= 0.5).astype(int)

acc = accuracy_score(y, oof_pred)

try:

auc = roc_auc_score(y, oof_prob)

except ValueError:

auc = float("nan")

brier = brier_score_loss(y, oof_prob)

cm = confusion_matrix(y, oof_pred).tolist()

return {

"oof_prob": oof_prob,

"oof_pred": oof_pred,

"acc": acc,

"auc": auc,

"brier": brier,

"cm": cm,

}

# ---------------------------------------------------------------------

# Optional plotting helpers (ROC and reliability)

# ---------------------------------------------------------------------

def plot_roc(y_true: np.ndarray, y_prob: np.ndarray, title: str, fname: str):

from sklearn.metrics import roc_curve, auc

fpr, tpr, _ = roc_curve(y_true, y_prob)

A = auc(fpr, tpr)

plt.figure()

plt.plot(fpr, tpr, label=f"AUC={A:.3f}")

plt.plot([0, 1], [0, 1], "k--")

plt.title(title)

plt.xlabel("False Positive Rate")

plt.ylabel("True Positive Rate")

plt.legend()

plt.savefig(fname, bbox_inches="tight")

plt.close()

def plot_reliability(y_true: np.ndarray, y_prob: np.ndarray, title: str, fname: str, n_bins: int = 10):

bins = np.linspace(0, 1, n_bins + 1)

idx = np.digitize(y_prob, bins) - 1

centers = 0.5 * (bins[:-1] + bins[1:])

obs = [y_true[idx == b].mean() if np.any(idx == b) else np.nan for b in range(n_bins)]

plt.figure()

plt.plot([0, 1], [0, 1], "k--", label="Perfect")

plt.plot(centers, obs, "o-", label="Observed")

plt.title(title)

plt.xlabel("Predicted probability")

plt.ylabel("Observed MRSA fraction")

plt.legend()

plt.savefig(fname, bbox_inches="tight")

plt.close()

# mrsa_audit_pipeline.py

# mrsa_audit_pipeline.py

"""

Audit pipeline for MRSA-vs-MSSA logistic model:

1) 36-case provenance (OOF prob, fold, TP/TN/FP/FN)

2) Fit final calibrated model on all 36, predict full 93-case cohort

3) Apply SA-PCR gating (display_prob=0, decision="No MSSA/MRSA" if PCR_SA_pos==0)

4) Save CSV/XLSX tables (+ optional OOF ROC & reliability)

Depends on mrsa_common.py:

- load_master_mrsa, get_sa_dataset, cv_calibrated_eval

- FEATURES, TARGET, ID_COL

- plot_roc, plot_reliability

- _make_calibrator (sklearn API compatible)

"""

from pathlib import Path

import json

import numpy as np

import pandas as pd

from sklearn.pipeline import Pipeline

from sklearn.impute import SimpleImputer

from sklearn.preprocessing import StandardScaler

from sklearn.linear_model import LogisticRegression

from sklearn.model_selection import StratifiedKFold

from mrsa_common import (

load_master_mrsa, get_sa_dataset, cv_calibrated_eval,

FEATURES, TARGET, ID_COL, plot_roc, plot_reliability, _make_calibrator

)

OUTDIR = Path("out_pipeline")

OUTDIR.mkdir(exist_ok=True)

def make_logreg_pipeline() -> Pipeline:

return Pipeline([

("imp", SimpleImputer(strategy="median")),

("sc", StandardScaler()),

("clf", LogisticRegression(max_iter=1000, class_weight="balanced")),

])

def build_provenance_table(gt_df: pd.DataFrame,

oof_prob: np.ndarray,

oof_pred_bin: np.ndarray,

fold_assign: np.ndarray) -> pd.DataFrame:

"""Assemble per-case provenance for the 36 AST-confirmed S. aureus positives."""

prov = gt_df[[ID_COL, "Culture_SA", "PCR_SA_call", "Ct_SA",

"PCR_mecA_call", "Ct_mecA", "Ct_CoNS",

"dCt_SA", "dCt_CoNS", "P_SA", "P_CoNS", TARGET]].copy()

prov["fold"] = fold_assign

prov["oof_prob"] = oof_prob

prov["oof_pred"] = np.where(oof_pred_bin == 1, "MRSA", "MSSA")

truth = (prov[TARGET] == "MRSA").astype(int).values

predb = (prov["oof_pred"] == "MRSA").astype(int).values

prov["result"] = np.where((predb == 1) & (truth == 1), "TP",

np.where((predb == 0) & (truth == 0), "TN",

np.where((predb == 1) & (truth == 0), "FP", "FN")))

return prov

def main():

# 1) Load engineered data and ground-truth cohort (36 culture+ SA with AST)

df = load_master_mrsa(Path("Master data_MRSA.xlsx"))

X, y, ids = get_sa_dataset(df)

gt = df[df["is_ground_truth"]].copy() # rows aligned with X,y

# 2) 5-fold OOF with calibration for logistic regression

pipe = make_logreg_pipeline()

res = cv_calibrated_eval(pipe, X, y, cv_splits=5, seed=42)

# Derive fold assignments (for transparency) with the same splitter

skf = StratifiedKFold(n_splits=5, shuffle=True, random_state=42)

fold_assign = np.zeros(len(y), dtype=int)

for f, (_, te) in enumerate(skf.split(X, y), start=1):

fold_assign[te] = f

# 36-case provenance

prov = build_provenance_table(gt, res["oof_prob"], res["oof_pred"], fold_assign)

prov_csv = OUTDIR / "provenance_36_cases.csv"

prov.to_csv(prov_csv, index=False)

# OOF summary metrics (for reporting)

summary_oof = {

"acc": float(res["acc"]),

"auc": float(res["auc"]),

"brier": float(res["brier"]),

"cm": res["cm"],

}

(OUTDIR / "summary_oof.json").write_text(json.dumps(summary_oof, indent=2))

# Optional OOF figures

plot_roc(y.values, res["oof_prob"], "ROC – Logistic (OOF)", str(OUTDIR / "logreg_oof_roc.png"))

plot_reliability(y.values, res["oof_prob"], "Reliability – Logistic (OOF)", str(OUTDIR / "logreg_oof_reliability.png"))

# 3) Fit final calibrated model on all 36, then predict full 93-case cohort

final_cal = _make_calibrator(make_logreg_pipeline(), method="sigmoid", cv=5)

final_cal.fit(X, y)

P_all = final_cal.predict_proba(df[FEATURES])[:, 1]

# 4) SA-PCR gating

display_prob = np.where(df["PCR_SA_pos"] == 1, P_all, 0.0)

decision = np.where(df["PCR_SA_pos"] == 1,

np.where(display_prob >= 0.5, "MRSA", "MSSA"),

"No MSSA/MRSA")

# 5) Save 93-case table (CSV + XLSX)

out_full = df[[ID_COL, "Culture_SA", "PCR_SA_call", "Ct_SA",

"PCR_mecA_call", "Ct_mecA", "Ct_CoNS",

"dCt_SA", "dCt_CoNS", "P_SA", "P_CoNS"]].copy()

out_full["calibrated_prob"] = P_all

out_full["display_prob"] = display_prob

out_full["decision"] = decision

full_csv = OUTDIR / "full_93_cases_gated.csv"

full_xlsx = OUTDIR / "full_93_cases_gated.xlsx"

out_full.to_csv(full_csv, index=False)

out_full.to_excel(full_xlsx, index=False)

print("Wrote:", prov_csv)

print("Wrote:", OUTDIR / "summary_oof.json")

print("Wrote:", full_csv)

print("Wrote:", full_xlsx)

print("Saved OOF ROC/reliability plots in", OUTDIR)

if __name__ == "__main__":

main()

# model_logreg.py

# model_logreg.py

import json

from pathlib import Path

from sklearn.pipeline import Pipeline

from sklearn.preprocessing import StandardScaler

from sklearn.linear_model import LogisticRegression

from sklearn.impute import SimpleImputer

from mrsa_common import load_master_mrsa, get_sa_dataset, cv_calibrated_eval, plot_roc, plot_reliability

OUTDIR = Path("out_logreg")

OUTDIR.mkdir(exist_ok=True)

def main():

# Load engineered data from the Master file

df = load_master_mrsa(Path("Master data_MRSA.xlsx"))

X, y, ids = get_sa_dataset(df)

pipe = Pipeline([

("imp", SimpleImputer(strategy="median")),

("sc", StandardScaler()),

("clf", LogisticRegression(max_iter=1000, class_weight="balanced")),

])

# 5-fold OOF with calibration

res = cv_calibrated_eval(pipe, X, y, cv_splits=5, seed=42)

# Save summary (keys aligned with compare_models.py)

summary = {

"model": "LogisticRegression",

"acc": float(res["acc"]),

"auc": float(res["auc"]),

"brier": float(res["brier"]),

"cm": res["cm"],

}

(OUTDIR / "summary.json").write_text(json.dumps(summary, indent=2))

# Optional figures

plot_roc(y.values, res["oof_prob"], "ROC – Logistic Regression", str(OUTDIR / "roc.png"))

plot_reliability(y.values, res["oof_prob"], "Reliability – Logistic Regression", str(OUTDIR / "reliability.png"))

print("LogReg ->", OUTDIR / "summary.json")

if __name__ == "__main__":

main()
